# The Variability of the Salivary Antimicrobial Peptide Profile: Impact of Lifestyle

**DOI:** 10.3390/ijms252111501

**Published:** 2024-10-26

**Authors:** Mariana Gallo, Elena Ferrari, Laura Giovati, Thelma A. Pertinhez, Lorenza Artesani, Stefania Conti, Tecla Ciociola

**Affiliations:** 1 Laboratory of Biochemistry and Metabolomics, Department of Medicine and Surgery, University of Parma, 43125 Parma, Italy; mariana.gallo@unipr.it (M.G.); elena.ferrari@unipr.it (E.F.); 2 Laboratory of Microbiology and Virology, Department of Medicine and Surgery, University of Parma, 43126 Parma, Italy; laura.giovati@unipr.it (L.G.); lorenza.artesani@unipr.it (L.A.); stefania.conti@unipr.it (S.C.); tecla.ciociola@unipr.it (T.C.); 3 Microbiome Research Hub, University of Parma, 43124 Parma, Italy

**Keywords:** saliva, antimicrobial peptides’ profiles, variability, lifestyle, biomarkers

## Abstract

Saliva is crucial in maintaining oral health; its composition reflects the body’s physiological and diseased state. Among salivary components, antimicrobial peptides (AMPs) stand out for their broad antimicrobial activities and role in modulating the oral microbiota and innate immune response. Local and systemic diseases can affect the levels of AMPs in saliva, making them attractive biomarkers. However, the large variability in their concentrations hampers their use in diagnostics. Knowledge of the various factors influencing the profile of salivary AMPs is essential for their use as biomarkers. Here, we examine how lifestyle factors such as physical activity, dietary supplementation, tobacco smoking, and psychological stress impact salivary AMP levels. By understanding these sources of variability, we can take a step forward in using AMPs for diagnostics and prognostics and develop new tailored and preventative approaches.

## 1. Introduction

**Figure 1:**
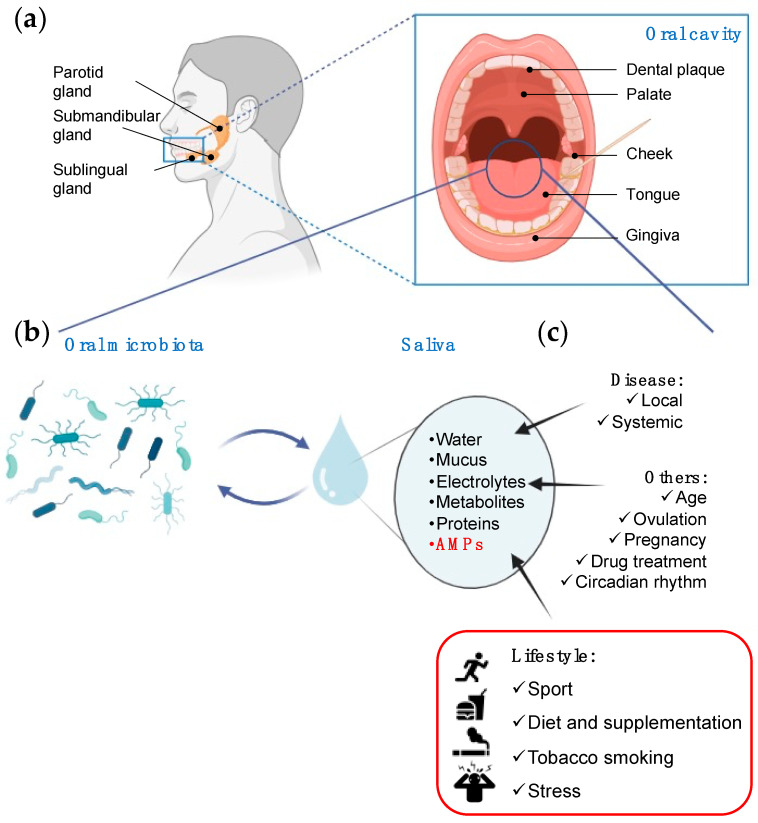
Origin and variable composition of saliva. (**a**) Salivary glands and oral cavity. (**b**) Oral microbiota and saliva composition exert a reciprocal influence. (**c**) Factors impacting on saliva composition. Disease, individual variability, and lifestyle influence the saliva composition, including the AMP profile. Created with BioRender.com.

The oral cavity is a complex ecosystem with many subsites, including tooth surfaces, gingiva, cheek, palate, tongue, and saliva (Figure 1a). Saliva is a fluid secreted into the mouth by salivary glands and composed of water, proteins, metabolites, electrolytes, and antimicrobial peptides (AMPs) [1]. Saliva functions involve lubricating the mouth, stabilizing oral pH, aiding teeth remineralization, preparing food for swallowing, starting digestion, and modulating growth and adherence of oral microorganisms to tooth surfaces [2]. Saliva is crucial for maintaining oral health, and, due to its composition and functions, it is considered the “mirror of the body,” reflecting virtually the entire spectrum of normal and disease states [3]. Because of the molecular exchange between blood and saliva, saliva is a good indicator of the plasma levels of various substances such as hormones or drugs. A correlation has been observed between serum and saliva concentrations of specific metabolites [4]. Therefore, saliva is an attractive fluid for diagnostic purposes. However, even if salivary biomarkers may provide information about local and systemic diseases [5], the large variability in saliva composition compared with blood or urine is one of the challenges for using saliva in diagnostics [4,6].

A microbiota is an ecological community of symbiotic and commensal microorganisms occupying a well-defined habitat [7]. The oral cavity provides an optimal environment for the growth of microorganisms because of the stable and appropriate temperature and humidity and the availability of nutrients (Figure 1b) [8]. It hosts more than 700 bacterial species, eukaryotes, archaea, and viruses, constituting the oral microbiota [9]. This is the second most complex human microbiota after the gut [10].

Salivary antimicrobial polypeptides are the first line of defense in the oral and gastrointestinal systems, ranging from large proteins like mucins to smaller cationic peptides like histatins and defensins [11]. The panel of salivary proteins involved in the immune defense system with direct and indirect antimicrobial activities includes mucins, secreted immunoglobulin A (sIgA), lactoferrin, cystatins, lactoperoxidase, and lysozyme. These proteins have different modes of action for their antimicrobial function. Mucins block the adhesion of certain microorganisms to oral surfaces; sIgA molecules are the most abundant antibodies at the mucosal surface; lactoferrin sequesters iron from microorganisms; cystatins inhibit the cysteine proteinases secreted by bacteria; lactoperoxidase produces hypothiocyanite, which inhibits important bacterial metabolic processes; and lysozyme catalyzes the degradation of the bacterial cell wall [10,11,12].

Salivary AMPs, such as histatins, defensins, cathelicidins, statherin, adrenomedullin, various neuropeptides, and the breakdown products of larger salivary proteins are typically amphipathic cationic peptides. These peptides differ significantly in sequence and structure [11,13] and protect against a broad spectrum of pathogenic microorganisms [14]. They bind to and interact with the microbial cell membranes, eventually leading to pathogen cell death through various mechanisms of action. Some AMPs cause cell membrane damage and permeation, disrupting cell morphology, while others have intracellular targets [14,15,16,17]. Interestingly, many salivary AMPs have additional proinflammatory and proapoptotic activities [18,19].

Local and systemic diseases can affect the salivary levels of AMPs and antimicrobial proteins [12], making them attractive biomarkers. However, disease is not the only factor influencing the expression profiles of AMPs and antimicrobial proteins, which also depend on individual conditions such as age, gender, and lifestyle. This last can lead to large individual variability in AMPs and antimicrobial protein concentrations, hampering their use in salivary diagnostics [20]. Antimicrobial proteins, mainly sIgA [21,22], lactoferrin, and lysozyme [23,24], have been measured to investigate changes in oral mucosal immunity in response to different stimuli. Less is known about the factors influencing salivary AMP levels. Nevertheless, we recognize that a thorough understanding of the various factors that affect AMP profiles and contribute to their variability in saliva composition is important to improve the use of saliva as a diagnostic fluid.

In this review, we will focus on the impact of lifestyle on salivary AMP concentrations (Figure 1c). We provide an introductory section on (i) how oral microbiota and salivary composition influence each other and (ii) the characteristics of salivary AMPs whose variations in concentration have been associated with specific diseases, underlining their potential use as biomarkers. The following section reviews the various studies documenting the impact of individual lifestyles on specific salivary AMP levels and comprehensively discusses their variations.

## 2. The Interplay Between Saliva and Microbiota

Saliva is key in maintaining a symbiotic relationship between the host and the oral microbiota [10]. It provides the oral microbiota with nutrients, and in return, the microorganisms exchange metabolites, proteins, and electrolytes with saliva, thereby influencing the saliva composition.

Saliva permits some microorganisms to colonize the oral cavity but also prevents extensive microbial colonization. In healthy conditions, saliva’s antimicrobial capacity dictates the milieu that will promote the establishment of certain bacterial species and prevent pathogens. This complex interplay is essential for sustaining a balanced and beneficial microbiota and, eventually, for maintaining oral and systemic health [10].

Salivary antimicrobial proteins and AMPs play an integral role in this process, regulating oral colonization and modulating the innate immune response to induce tolerance to commensal organisms [12,25].

## 3. Salivary AMPs

Gorr et al. reported the identification of 45 AMPs and antimicrobial proteins in saliva but suggested that additional AMPs may be present [26]. Grant and collaborators have measured 63 AMPs and proteins using multiplex selective reaction monitoring mass spectrometry in saliva samples of 41 adult subjects [25]. To date, the human salivary databank (www.salivaryproteome.org, accessed on 31 July 2024) contains 77 salivary protein sequences with antimicrobial activity. Salivary AMPs (less than 50–60 amino acids) are synthesized mainly by salivary acini and ducts, oral epithelia, and immune cells but can also be the product of the hydrolysis of larger proteins. The most abundant peptide fragments present in saliva have been reported to originate from proline-rich proteins (PRP), statherin, and histatins [27]. The most relevant AMPs expressed in the oral cavity, and whose levels have been measured in clinical studies, are listed in Table 1 ([2,11,28,29] and references therein).

## 4. Salivary AMPs as Potential Biomarkers

The health of the oral cavity is influenced by several factors, including the presence of specific microorganisms and a range of extrinsic and intrinsic factors that affect oral conditions and saliva composition. The expression of AMPs can be constitutive or stimulated by infections and/or inflammatory stimuli, such as proinflammatory cytokines or bacteria [31]. Therefore, salivary AMP levels are altered in numerous oral and systemic diseases (Table 2). Accordingly, salivary AMPs have been proposed as potential biomarkers, especially for oral pathologies [26,32]. However, the levels of AMPs in saliva are highly dynamic and result from diverse circumstances, leading to significant individual variations, which presents a challenge in the interpretation of the clinical data [33,34,35]. Although the salivary levels of AMPs can be distinguished between healthy and disease samples using statistical methods, the considerable variability in AMP levels makes differential diagnosis difficult [20].

## 5. The Effect of Lifestyle on AMP Expression Profiles

In addition to pathological processes and diseases, salivary composition, including the levels of AMPs, is influenced by factors related to individual characteristics, such as sex [54], age [55], circadian rhythm [56], pregnancy, and hormonal status [57,58].

To assess and improve the predictive and diagnostic capabilities of AMP levels, we reviewed the literature to identify any potential confounding factors that may alter the salivary profile of AMPs in daily life. We focused on randomized controlled trials published in the last fifteen years using PubMed, Google Scholar, Web of Science, and Academia.edu databases with the terms “saliva” and “antimicrobial peptides” combined with a particular lifestyle, such as “physical activity” or “sport”; “diet”, “nutrient”, or “diet supplementation”; “smoke” or “tobacco”; “stress” or “psychological stress”. We searched for the impact of a single lifestyle on the concentrations of specific salivary AMPs.

It is important to note that most of the reviewed studies employed unstimulated whole saliva and measured the concentrations of antimicrobial peptides using ELISA assays. As indicated in the corresponding tables, only a few studies used stimulated whole saliva as a matrix or mass spectrometry as the analytical method. Given the consistency in sample type and method of AMP quantification, the results of most of the reviewed studies should be comparable. In the following paragraphs, we summarize the results from various studies, analyzing each feature separately.

### 5.1. Physical Activity

Intense physical activity induces widespread responses in cells, tissues, and organs to meet the increased demands for energy and oxygen. Physiological and biochemical processes are triggered to adapt the body to these requirements [59]. Strong signatures of molecular regulation during physical activity have been reported in different biofluids, including saliva [60,61]. When these processes fail to compensate for the demands of physical stress, the consequences are exhaustion, diminished performance, and increased susceptibility to disease. For instance, heavy training is associated with an increased risk of respiratory infection [62,63], and, in physically active individuals, low sIgA secretion correlates with the development of upper respiratory tract infections [64]. AMPs play an important role in this context.

In addition to their direct effect on microorganisms, AMPs exert their protective effect via immunomodulatory activities by recruiting immune cells, inducing cytokine secretion, and repairing damaged epithelia [65]. AMPs are involved in leukocyte recruitment, chemotaxis stimulation, pro- and anti-inflammatory cytokine induction, endotoxin neutralization, and activation and differentiation of immune cell lines [66].

Several studies have reported the effect of physical activity on the levels of specific salivary AMPs. Table 3 summarizes some of the most recent and relevant findings.

Physical activity can be chronic or acute. In the latter case, it can be high, medium, low, or increasing intensity. The effects of a prolonged cycle ergometer test (2.5 h at 60% maximal oxygen consumption, VO_2max_) performed on 12 active men on salivary sIgA, human neutrophil peptides 1–3 (hNP 1–3), and LL-37 were investigated by Davison et al. [67]. Results showed that, while sIgA secretion remained unaltered, hNP 1–3 and LL-37 salivary concentrations were significantly increased in response to acute exercise. Similarly, 60 min of exercise on a cycle ergometer at 75% VO_2max_ caused a transient increase in the oral human β-defensin-2 (hβD-2) and LL-37 levels but a reduction in sIgA in 10 men [68]. Gillum et al. observed an increase in the salivary levels of LL-37 and hNP 1–3, along with sIgA, lactoferrin, and lysozyme in participants after 45 min of running at 75% VO_2max_ [69]. In a more recent multi-omics study of biofluids collected from 11 wildland firefighters before and after 45 min of intense exercise, Nakayasu et al. found a decrease in three proinflammatory cytokines and an increase in eight antimicrobial proteins and AMPs in saliva samples [61]. The AMPs and antimicrobial proteins that were up-regulated after physical activity were dermcidin, cystatins (S, SN, SA, C, D), hβD-1, and histatin 1. A reduction in specific oral microbes correlated with increased salivary AMPs. Kunz et al. observed an increase in the concentration and/or secretion of hNP 1–3 and LL-37, lysozyme, lactoferrin, and sIgA after exercise (three × 30 min trials with different workloads) [70].

In all these studies, the levels of salivary AMPs were transiently increased after an acute physical exercise session. This increase in AMPs is consistent with a compensatory mechanism to improve host defense in the immediate post-exercise period. Differences in the participants’ training status, absolute intensity, and duration of the training session may account for the contrasting findings regarding lysozyme and sIgA concentrations. As for lysozyme, the response may have been too low, or the physical activity not intense enough to induce variations; as for sIgA, a marker of mucosal immune defense, the divergent results suggest that there may be factors controlling IgA levels that are not yet well understood.

Kunz et al. detected lower basal levels of hNP 1–3 (and lactoferrin) in high- vs. low-fit cyclists. At the same time, LL-37, lysozyme, and sIgA concentrations were not significantly different between the two groups [70].

Lower levels of hβD-2 and LL-37 were found in marathon runners compared with sedentary controls [71]. Interestingly, the authors also found that upper respiratory tract infections were negatively associated with salivary hβD-2 and LL-37 concentrations in marathon runners and sedentary controls. Prolonged intense or chronic physical exercise appears to be associated with reduced concentrations of salivary AMPs, leading to reduced oral respiratory mucosal immunity, which may partly account for the increased incidence of upper respiratory tract symptoms in athletes. In line with this hypothesis, lactoferrin levels were lower in elite rowers than in non-exercising volunteers [33] and basketball players over a competitive training season than in a resting period [72]. Variations in sIgA levels due to chronic exercise contrast in different studies, supporting more complex mechanisms modulating sIgA levels in saliva [70,73,74]. In some cases, lysozyme salivary levels are invariant in athletes compared with sedentary controls [33,70], while in others, they are decreased [72], suggesting a less sensitive variation.

### 5.2. Diet Supplementation

Supplementation with micronutrients (minerals and vitamins) and macronutrients (carbohydrates, proteins, and fats) influences the expression of endogenous AMPs, as evidenced by several studies in cell cultures and animals (revised by [75,76]). It has been proposed that diet and/or supplementation may modulate immune function by inducing the local transcription and expression of salivary AMPs [76]. Nutritional supplementation has been used to compensate for the decline in innate mucosal immunity associated with increased upper respiratory illness in athletes [77]. However, clinical studies in humans demonstrating variations in AMPs due to ingesting specific nutrients are limited. These studies are summarized in Table 4 and classified according to the type of nutrient. The effect of the supplementation on mucosal immunity is often quantified by measuring the changes in AMP levels in a cohort of subjects exposed to the supplemented diet compared with subjects not exposed to supplementation.

#### 5.2.1. Vitamin D

Vitamin D is critical for calcium homeostasis and bone mineralization. Vitamin D status is related to markers of innate mucosal immunity [75] and is acknowledged to improve innate immunity by enhancing the expression of AMPs in epithelial cells [89]. The immunomodulatory role of vitamin D may become increasingly important during periods of elevated stress. In a pioneering study, He et al. verified that plasmatic vitamin D levels correlated with the incidence of upper respiratory tract illness episodes in athletes during winter training. They also found a significant correlation between the salivary sIgA secretion rates and plasmatic vitamin D concentrations but no significant differences in salivary lactoferrin and lysozyme concentrations and secretion rates [90]. A successive study found that the secretion rates of sIgA and LL-37 were significantly increased in vitamin D_3_-supplemented vs. non-supplemented athletes [78]. Likewise, daily vitamin D supplementation (vitamin D_3_ and calcium) enhanced sIgA and LL-37 secretion rates in Marine Corps recruits undergoing 12 weeks of basic military training [79].

Vitamin D insufficiency has been strongly associated with dental caries [91]. Serum vitamin D deficiency was also related to an increased caries experience in women, and salivary LL-37 levels correlated with serum vitamin D concentration [80]. Therefore, vitamin D supplementation has been proposed to reduce the risk of dental caries, especially in children. In a study conducted on a cohort of 377 volunteers classified as caries-free or caries-active, the salivary vitamin D statistically decreased with the increasing severity of caries, but, in this case, the salivary LL-37 concentration was not significantly different between caries-active and caries-free groups [81]. In a study of patients with recurrent aphthous stomatitis, Bahramian et al. attributed the lack of statistical difference between salivary vitamin D in the case and control groups to the lower vitamin D levels in saliva compared with serum. This may explain why salivary vitamin D did not correlate with salivary LL-37 levels in Nireeksha’s study [81].

#### 5.2.2. Bovine Colostrum, Fermented Milk, and Probiotics

Bovine colostrum (BC), the milk that cows produce in the first few days after giving birth, is rich in bioactive components that play significant roles in the immune system. The higher concentrations of these bioactive constituents in BC have been suggested to benefit human immune health [92]. Oral supplementation with BC has been shown to enhance immunity in athletes. Several studies on sIgA, lactoferrin, and lysozyme show that the beneficial effects of BC are not reflected in the increase in lactoferrin or lysozyme, while the results for sIgA are inconclusive [93]. Studies investigating the possible changes in salivary AMP levels induced by BC supplementation are missing.

Probiotics and fermented milk are another line of diet supplementation used to combat the increased incidence of upper respiratory tract infections in athletes. *Lactobacillus* spp. has been shown to provide protective effects on the incidence of respiratory and gastrointestinal tract symptoms [94,95]. Vaisberg et al. investigated the effect of daily intake of fermented milk in modulating the immune response in marathon runners [82]. They found lower salivary levels of salivary sIgA and hNP 1 immediately post-marathon in the placebo compared with the fermented milk intake group. In contrast, lactoferrin, LL-37, and lysozyme levels were not statistically different when comparing both groups after the marathon.

On the other hand, the administration of probiotics is also considered a potential strategy to improve or maintain oral health. The elevation of salivary hNP 1–3 levels by probiotic milk containing *L. paracasei* was detected in teenagers with good oral health [83] and preschool children with severe caries [84]. Further studies are needed to understand the underlying mechanisms of increased salivary hNP 1–3 production induced by probiotics. A preventive role of salivary hNP 1–3 against dental caries has previously been suggested [20]. Another study demonstrated that a regular intake of *L. rhamnosus*-supplemented milk decreased the occurrence of caries and salivary levels of hβD-3 in preschool children [85]. The authors hypothesized that the effect of probiotics on hβD-3 levels may be associated with a decreased caries prevalence and the reestablishment of healthy oral microbiota. They proposed salivary hβD-3 as a marker of oral tissue homeostasis.

#### 5.2.3. Carbohydrates and Proteins

Another nutritional strategy to counter exercise-induced immune dysfunction in athletes is combining carbohydrates with high-quality protein sources. Naclerio et al. compared the effect of carbohydrate- vs. protein-rich diets on salivary AMP levels in athletes. They investigated the effects of ingesting hydrolyzed beef protein, whey protein, and carbohydrates on salivary hNP 1–3 levels (as a reference of humoral immunity) following an 8-week resistance training program in college athletes [87]. They measured decreased levels and hNP 1–3 secretion rates from baseline to post-workout only for the beef condition. In another study, they analyzed the long-term effects of ingesting hydrolyzed beef protein vs. carbohydrate during ten weeks of endurance training in master-aged triathletes by measuring salivary hNP 1–3 levels before and after an incremental endurance test to exhaustion, both before and after the intervention [86]. Again, there were no differences between the baseline in the two groups. However, although the hNP 1–3 secretion rate after the post-intervention incremental test decreased in both groups, the hNP 1–3 concentration reduction was greater in the protein group. Both studies concluded that protein alone, without carbohydrates, may not be as effective as carbohydrate alone in attenuating negative long-term changes in hNP 1–3, which is considered an immunological marker.

#### 5.2.4. Vitamin A

Retinoic acid (RA), an active vitamin A derivative, has important roles in vision, cell development, skin and mucous membranes maintenance, immune function, and reproductive development [96]. In addition to being involved in various physiological processes, RA also affects the expression of AMPs [97]. Reduced salivary levels of hβD-2, but not hβD-1 or hβD-3, were detected in systemic RA users compared with non-user controls [88]. According to the authors, the observed changes in hβD-2 levels may be associated with RA-related inflammatory processes.

### 5.3. Tobacco Smoking

Tobacco smoking causes structural changes in the respiratory tract, leading to a substantial decrease in the host immune response, making it a risk factor for a wide range of diseases and infections [98]. Table 5 summarizes the main findings related to the effect of tobacco smoke on salivary AMPs.

Periodontal diseases comprise inflammatory conditions with diverse levels of severity affecting the structures supporting the teeth. They are produced by a dysbiosis of the oral microbiota, which, interacting with the immune host defenses, leads to inflammation and disease [100]. Tobacco is the most important avoidable risk factor in the incidence and progression of periodontal diseases [101]. There is evidence of an association between periodontal disease and increased LL-37 expression in saliva [36]. To evaluate the performance of LL-37 as a potential biomarker for periodontitis in the presence/absence of risk factors such as tobacco smoking, Kzar et al. carried out a study with 160 participants comprising 80 healthy subjects (40 smokers and 40 non-smokers) and 80 subjects with periodontitis (40 smokers and 40 non-smokers) [40]. They found that LL-37 was higher in periodontal than healthy subjects in non-smokers. However, LL-37 levels in smokers were lower than in non-smokers. Even though tobacco smoke reduced LL-37 salivary levels, LL-37 was still able to statistically distinguish between health and periodontal disease, regardless of the smoking status of the participants. In agreement, Takeuchi et al. found a negative correlation between salivary LL-37 and cotinine, a nicotine metabolite and biomarker of active tobacco smoking, in patients with periodontitis [36]. Neutrophils are known to be impaired by tobacco, which is expected to affect the production of LL-37. However, the underlying mechanisms remain unknown [102]. Most of the harmful effects of tobacco are due to first-hand (active) as much as second-hand (passive) smoke [103,104,105]. A study of 180 children, either exposed or not to passive tobacco smoking, demonstrated that passive smoking reduced salivary LL-37 concentrations in children [99], in line with Kzar’s and Takeuchi’s results.

In another study involving 41 volunteers grouped into healthy non-smokers, non-smokers with periodontal disease, healthy tobacco smokers, and tobacco smokers with periodontal disease, Grant et al. profiled 63 salivary AMPs and antimicrobial proteins [25]. They did not find differences in the levels of adrenomedullin, dermcidin, several hβDs, hNP 1, cathelicidin (precursor of LL-37), and neuropeptide Y in healthy smokers and non-smokers. The smaller number of participants, the fact that they measured the precursor cathelicidin instead of LL-37, or that they used stimulated rather than unstimulated saliva may explain the contrasting results compared with the studies by Kzar and Karsiyaka Hendek et al. [40,99]. Grant et al. also found a statistical increase in cathelicidin associated with periodontitis in non-smoking participants. However, no differences were measured for this peptide in periodontitis associated with tobacco smoking [25]. In this study, probably the decrease in cathelicidin associated with tobacco smoking likely compensated for the increase associated with periodontitis.

### 5.4. Psychological Stress

Psychological stress activates the hypothalamic–pituitary–adrenal (HPA) axis, causing the release of corticoid hormones and catecholamines into the circulation and down-regulating the innate immune response [106]. The biological changes associated with stresses have been depicted as risk factors for oral diseases, including periodontitis, in which anerobic bacteria colonize the subgingival area [107]. Accordingly, stressful conditions are expected to influence the AMP levels in saliva. Table 6 summarizes some studies on the effects of psychological stress on salivary AMPs.

As psychological stress has been proposed to inhibit the production of β-defensins [112], Forte et al. investigated the association between stress and hβD-2 and hβD-3 salivary levels [108]. Seventy-five volunteers were classified as stressed or not stressed based on a psychological evaluation using a validated questionnaire. The levels of both β-defensins were not statistically different in both groups.

Recognizing that yoga reduces psychological stress, Eda et al. investigated the effect of yoga on the salivary levels of hβD-2, finding an increase in hβD-2 in a cohort of 15 volunteers after 90 min of yoga stretching [109]. The authors suggested that stress reduction through yoga may stimulate hβD-2 production, possibly enhancing immune function. The different approaches and conditions may explain the contrasting results of the studies by Forte et al. [108] and Eda et al. [109].

Some studies have reported evidence of immune down-regulation in response to spaceflight missions [113,114]. Considering that spatial missions are a source of psychological stress due to mission goals, extended periods of isolation, disturbances of the circadian rhythm, microgravity, noise, etc., Agha et al. investigated whether salivary levels of antimicrobial proteins and AMPs were altered in response to long-term missions [110]. When comparing International Space Station crew members to ground-based control subjects, the first exhibited higher salivary levels of sIgA, lysozyme, and LL-37. In contrast, no differences were observed in lactoferrin and hNP 1–3. In addition, crew members embarking on their first space mission had lower levels of salivary sIgA but higher levels of lysozyme and LL-37, while no differences in lactoferrin and hNP 1–3 were observed during and after the mission compared with the veterans. The fact that these changes correlated with biomarkers of the sympathetic HPA axis activity supports the view that immune dysregulation in spaceflight is a stress-related phenomenon and that stress-relieving actions are needed to preserve the immunity of crew members in prolonged space missions.

Gillum et al. investigated the effect of acute sleep deprivation, which can lead to increased psychological stress, on salivary AMP levels after exercise [69]. In a cohort of eight volunteers, LL-37 and hNP 1–3 levels were transiently increased after 45 min of running at 75% VO_2max_. However, sleep loss did not affect the concentration or secretion rate of these AMPs before or in response to exercise. This result is consistent with acute sleep loss not affecting stress hormones in humans [115].

Neuropeptide Y has been identified as one of the main neurotransmitters playing an important role in stress resilience [116]. A study has investigated the relationship between the salivary levels of neuropeptide Y and academic stress during tests in a cohort of undergraduate students. Saliva samples were collected from 44 students before and after an exam. Salivary cortisol, neuropeptide Y, and IL-1 β levels were significantly increased after the test [111].

## 6. Discussion

We have addressed physical activity, nutrient supplementation, tobacco smoking, and psychological stress to better understand the underlying factors that account for AMP variability.

Most of the studies examined in this review utilize ELISA assays to quantify the AMPs in unstimulated whole saliva samples. The consistency in sample type and analytical approach supports the comparability of results across studies.

The randomized controlled trials employed in the studies included different subject groups. Some studies assess the same cohort before and after interventions such as exercise, nutrient supplementation, or a stressful situation, while others compare groups that are homogeneous in terms of age and gender. For instance, studies analyzing the impact of acute physical activity on salivary AMPmeasure changes before and at different times after intense exercise; instead, when analyzing the effect of chronic physical exercise, a comparison is made between two groups: low- and high-fit or inactive and active volunteers (Table 3). These trials vary in exercise intensity and involve a limited number of volunteers (between 8 and 20). Despite these limitations, the results tend to be consistent across studies.

In contrast, the reviewed studies involving dietary supplementation tend to involve larger cohorts, ranging from 16 to 373 participants (Table 4). These studies focus on changes in AMP levels before and after supplementation. However, as supplementation periods are often quite long, other behavioral changes over time may also influence the results. Comparisons between supplemented and placebo groups are also common. Notably, given the intrinsic variability of the population, the nutrient dose must be sufficient to produce observable significant changes. This consideration also applies to LL-37 variations due to tobacco smoking, both active and passive, where exposure should align with AMP changes in cohorts from 41 to 160 subjects (Table 5). The assessment of psychological stress is particularly challenging because of its multiple causes and effects, making it difficult to define appropriate cohorts.

Salivary AMP levels are regulated by the parasympathetic and sympathetic nervous systems, which control saliva flow rate and protein secretion, and fitness level is a major determinant of exercise-induced changes in AMP levels [21,70]. The effect of physical activity on salivary AMP concentrations depends on the duration and intensity of the exercise performed. Acute exercise tends to induce a transient increase in salivary AMP levels (α- and β-defensins, histatins, hNP 1–3, and LL-37), and fitness affects acute exercise response [70]. The increase in salivary AMPs correlates to a decrease in inflammatory markers, probably to improve respiratory function under physical stress conditions [61]. This can be part of a compensatory mechanism to increase immune surveillance under increased susceptibility to infection. In contrast, chronic exercise may induce lower basal levels of some specific antimicrobial proteins and AMPs (such as lactoferrin, defensins, and LL-37). The reduced levels of AMPs in subjects performing prolonged endurance exercise have been correlated with increased levels of salivary cortisol, possibly caused by the physical stress of exercise [68,71,72]. Increased cortisol may cause immunosuppression during strenuous exercise, and a subtle balance seems to exist between mucosal and adaptive immune responses. Although limited, the available studies suggest that salivary AMPs may serve as more accurate indicators of the mucosal immune response to prolonged and acute exercise than antimicrobial proteins such as sIgA, lysozyme, and lactoferrin. It appears that the salivary levels of these proteins are less affected by physical activity, indicating that different mechanisms regulate their expression.

Dietary supplementation with specific nutrients is a potential strategy to modulate immune function. It counteracts athletes’ declines in innate mucosal immunity [76,117]. Dietary supplementation based on vitamin D, bovine colostrum, fermented milk, probiotics, and diets rich in carbohydrates or proteins has been tested. The effects of different types of dietary supplements and study designs lead to changes in specific salivary AMP levels or secretion rates, e.g., vitamin D is associated with LL-37; fermented milk, probiotics, and carbohydrate supplementation with hNP 1–3; and vitamin A with hβD-2. The outcomes regarding AMP expression profiles depend on the specific study design employed.

The evidence suggests that tobacco smoking has an inhibitory effect on the expression of LL-37 in saliva [36], which is likely due to the damage of neutrophils, the primary source of LL-37, caused by tobacco products [102]. In addition, tobacco use represents the most important risk factor for periodontitis [101]. Periodontal lesions exhibit a marked increase in neutrophils, with elevated levels of LL-37 in saliva observed in periodontitis [118]. This evidence supports the proposal that LL-37 may be an early marker of inflamed tissues [119]. However, the reduction of LL-37 induced by tobacco smoking could mask the increase in salivary LL-37 levels observed in patients with periodontitis, thus hampering the utilization of salivary LL-37 as a periodontitis biomarker [36,40]. Further investigation is required to elucidate the impact of tobacco smoking on LL-37 salivary levels in patients with periodontitis to address this potential issue.

Psychological stress and some salivary peptides (LL-37, neuropeptide Y, and hβDs) correlate via the HPA axis and glucocorticoid modulation. It should be noted, however, that psychological stressors can be of different origins. Stress can be acute, prolonged, or chronic; the same stressful circumstance may induce diverse individual responses. Consequently, measuring and classifying psychological stress and quantifying its direct effects present significant challenges. Further research is required, including a rigorous experimental design to avoid confounding factors and to explore the underlying mechanisms of psychological stress and salivary AMPs.

Considering the emerging role of saliva as a diagnostic fluid and the observed variations in AMP levels caused by numerous pathologies (Table 2), the use of salivary AMPs as biomarkers is promising. However, the number of studies addressing the causes underlying the intrinsic variability of AMP profiles, crucial for their clinical use, is rather limited. To the best of our knowledge, the impact of several lifestyle-related habits, such as alcohol and drug consumption, unhealthy diets, poor oral hygiene, depression, etc., on salivary AMP profiles has not yet been investigated. In addition, information about the possible interaction of multiple factors influencing salivary AMP levels is still missing. Conducting studies with larger and more well-defined groups of volunteers and using standardized protocols for collecting saliva samples and measuring AMP levels may help to better interpret the relative variations of different AMPs in response to specific stimuli. Furthermore, these studies should assess the levels of a larger number of AMPs. Finally, investigations pointing to a better understanding of the molecular mechanisms underlying the regulation of AMP expression in saliva are essential to predicting and rationalizing the observed variations.

## 7. Concluding Remarks

**Figure 2:**
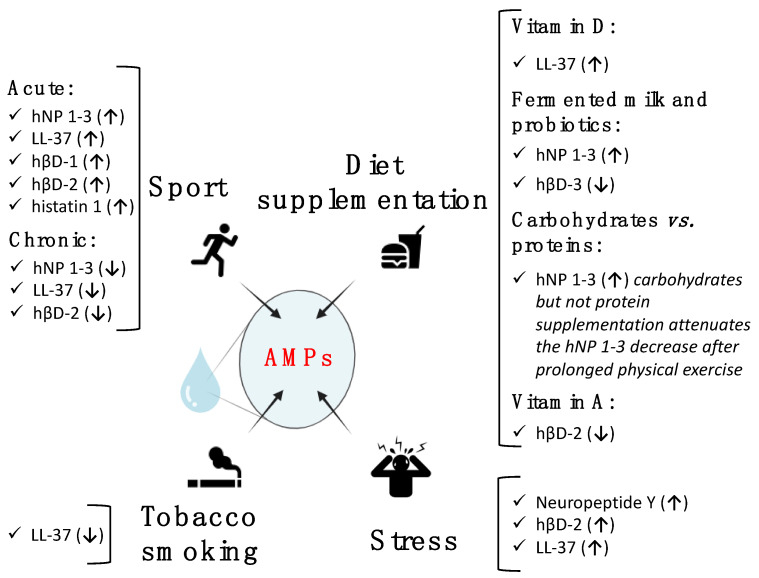
Summary of the main AMP variations induced by different lifestyles. Created with https://app.biorender.com/ (accessed on 31 July 2024).

This study aimed to gain insight into the various factors that influence the levels of salivary AMPs and contribute to the considerable variability that limits their clinical utility as biomarkers. We focused on the impact of some lifestyle features, summarized in Figure 2. The main findings are as follows:

- Physical activity: Acute physical exercise tends to temporarily increase salivary AMP levels, while prolonged physical activity seems to be associated with a reduction of them. This may be associated with the increased incidence of upper respiratory tract symptoms in athletes.
- Tobacco smoking: It induces a decrease in LL-37. This decrease could mask the increase in LL-37 correlated with periodontal diseases in smokers.
- Diet supplementation: Different supplements are associated with variations in specific salivary AMPs. For instance, vitamin D provokes an increase in LL-37 and vitamin A and a decrease in hβD-2; fermented milk decreases hβD-3 and increases hNPs (as in carbohydrate-supplemented diets).
- Psychological stress: it seems to increase some AMPs in saliva, though more studies are needed to confirm this trend.

Further research is needed to understand the molecular mechanisms involved in the expression and regulation of AMP levels in saliva and to investigate additional lifestyle habits. More comprehensive studies to enhance our understanding of salivary AMP profiles and the factors that influence them will facilitate the utilization of AMPs as indicators for disease prevention and diagnosis. They may also prove valuable in identifying and developing AMPs with therapeutic potential.

## Figures and Tables

**Table 1:** Main characteristics of the major salivary AMPs.

AMP	Structural Characteristics	Produced by	Biological Activity	Average Concentration (μg/mL) ^1^	References
**Histatins** (Histatin 1, 3, 5) and **proteolytic fragments**	7–38 AA, cationic, histidine-rich	Salivary gland and ductus	Bind metal ions Regulate hemostasis **Predominantly antifungal**	Histatin 1: 10.1 (parotid), 34.7 (SM/SL) Histatin 3: 7.3 (parotid), 10.2 (SM/SL)	[2,11,28,29]
**α-Defensins, human neutrophil peptides** (hNPs 1–4)	29–35 AA, cationic, cysteine-rich, three intramolecular S-S bonds (I-VI, II-IV, III-V), β-sheet fold	Neutrophils Gingival sulcus Inflammation site Salivary gland cells	Immunomodulation **Antibacterial** (Gram+ and Gram-) **Antifungal** **Antiviral** (HIV and HSV)	hNP-1: 8.6 hNP-2: 5.6 hNP-3: 0–2.7	[2,11,28,29]
**β-Defensins** (hβD-1, hβD-2, and hβD-3)	38–42 AA, cationic, cysteine-rich, three intramolecular S-S bonds (I-V, II-IV, III-VI), β-sheet fold	Epithelial cells Salivary duct	Induce TLR and PAR signalling and activate dendritic cells Help in tissue repair **Antibacterial** (Gram+ and Gram-) **Antiviral** (HIV) **Antifungal**	hβD-1: 0.15 hβD-2: 0.15 hβD-3: 0.31	[2,11,28,29]
**Cathelicidins** (LL-37)	C-terminal region of the hCAP-18, 37 AA, cationic, no cysteine, *α*-helical conformation	Neutrophils, monocytes, T cells Gingival sulcus Salivary gland and ducts	Chemo-attractant to immune cells **Primarily antibacterial** (Gram+ and Gram-) **Antifungal** **Antiviral** (HIV) **Antiparasitic**	1.6	[2,11,29]
**Statherin**	Phosphoprotein, 43 AA	Salivary gland cells	Inhibit calcium phosphate precipitation **Antibacterial** (anaerobic Gram-) **Antifungal**	26.5	[2,11,28,29]
**Adrenomedullin**	52 AA, cationic, one S-S bond	Epithelia	Vasodilator **Antibacterial** (Gram+ and Gram-)	0.06	[2,11,29]
**Neuropeptides** (substance P, neurokinin A, calcitonin gene-related peptide, neuropeptide Y, and vasoactive intestinal polypeptide)	10–37 AA, cationic	Salivary gland cells	Regulate stress responses and the salivary secretory mechanisms Concentration in saliva < MIC **Antibacterial** (Gram+ and Gram-) **Antifungal**	substance P: 7.5 × 10^−6^ neuropeptide Y: 41.4 × 10^−6^ calcitonin gene-related peptide: 23.5 × 10^−6^ vasoactive intestinal polypeptide: 39.9 × 10^−6^	[11,29]
**Fragments from PRPs** (P-B1, P-B, and BPLP)	Different fragments derived from PRPs	Parotid and submandibular glands	**Antibacterial** **Antiviral**	-	[2,11]
**Dermcidin**	Protein, 110 AA, processed to generate a 48 AA peptide with partial helical conformation	Striated cells in salivary glands	**Antibacterial** (Gram+ and Gram-) **Antifungal**	0.45	[30]

^1^ Concentrations refer to whole saliva unless otherwise indicated. All peptide concentrations are reported in [11], except for the protein dermcidin [30]. AA—amino acids; SM/SL—submandibular/sublingual glands; S-S—disulfide; hNP—human neutrophil peptide; HIV—human immunodeficiency virus; HSV—herpes simplex virus; hβD—human β-defensin; TLR—Toll-like receptor; PARs—proteinase-activated receptors; hCAP-18—human cationic antimicrobial protein; MIC—minimum inhibitory concentration; PRP—proline-rich protein; BPLP—basic proline-rich lacrimal protein; P-B—polymerase basic protein.

**Table 2:** Diseases associated with changes in salivary AMP levels.

Pathology		Changes in Salivary AMP Levels ^1^	References
**Oral**	**Periodontitis**	hβD-1, hβD-2, hβD-3 (↑) hNP 1 (↑) LL-37 (↑)	[25,36,37,38,39,40]
	**Infections**	Statherin (↑)	[41]
	**Candidiasis**	hβD-1, hβD-2 (↓)	[42]
	**Caries**	hNP 1–3 (↓) hβD-3 (↓) Histatin 5 (↓) LL-37 (↓) PPR IB-4 (↑) Statherin (↑)	[20,41,43,44,45]
	**Head and neck squamous cell carcinoma**	Histatin 1 (↑)	[46]
**Systemic**	**Alzheimer disease**	hNPs 1–4 (↑) Statherin (↑) Histatin 1 (↑)	[47]
	**Sjögren syndrome**	hβD-1, hβD-2 (↑)	[48]
	**Type 2 diabetes mellitus**	hβD-1 (↑) LL-37 (↑)	[39]
	**HIV infection**	hβD-1, hβD-2 (↑)	[49,50]
	**COVID-19**	hNP 1, hNP 3 (↑) hβD-3 (↑)	[51]
	**Long COVID-19**	Histatin 5 (↓)	[52]
	**Autoimmune hepatitis**	Histatins 3, 5, 6 (↑) Statherin (↑)	[53]

^1^ ↑, ↓—increase or decrease in salivary AMP levels (disease vs. health); concentrations refer to whole saliva. hβD—human β-defensin; hNP—human neutrophil peptide; PRP—proline-rich protein; HIV—human immunodeficiency virus.

**Table 3:** Influence of physical activity on salivary AMP levels.

Participants and Study Design	Observed Changes in Salivary AMP Levels ^1^	References
12 M (24 ± 8 y), 2.5 h of exercise on a cycle ergometer at 60% VO_2max_. Saliva sampling: immediately before and after exercise	Post- vs. pre-exercise: sIgA (=) **hNP 1–3** (**↑**) **LL-37** (**↑**)	Davison et al., *Eur. J. Appl. Physiol.* 2009 [67]
10 M (23 ± 3 y), 60 min of exercise on a cycle ergometer at 75% VO_2max_. Saliva sampling: during exercise and resting sessions at t_0_, 60, 120, and 180 min	During and post- vs. pre-exercise: **LL-37** (**↑**) **hβD-2** (**↑**) sIgA (↓)	Usui et al., *Eur. J. Appl. Physiol.* 2011 [68]
4 M and 4 F (23 ± 2 y) completed 2 exercise trials (45 min of running at 75% VO_2max_). Saliva sampling: before, immediately after, and 1 h after exercise	Post (1 h)- vs. pre-exercise: **hNP 1–3** (**↑**) sIgA (↑) Lysozyme (↑) Lactoferrin (↑) **LL-37** (**↑**)	Gillum et al., *J. Strength Cond. Res.* 2015 [69]
11 M (25 ± 3 y) wildland firefighters. Saliva sampling: before and immediately after 45 min of intense exercise regimen	Post- vs. pre-exercise: Lysozyme (=) **Dermcidin** (**↑**) Cystatins: S, SN, SA, C, and D (↑) **hβD-1** (**↑**) **Histatin 1** (**↑**)	Nakayasu et al., *Mil. Med. Res.* 2023 [61]
17 experienced cyclists (31 ± 5 y): 9 high-fit (6 M, 3 F) and 8 low-fit (7 M, 1F) completed three × 30 min exercise trials at varying workloads. Saliva sampling: before and immediately after exercise	Post- vs. pre-exercise: **hNP 1–3** (**↑**) sIgA (↑) Lysozyme (↑) Lactoferrin (↑) **LL-37** (**↑**) (increases were higher for high- vs. low-fit) High- vs. low-fit cyclists: **hNP 1–3** (**↓**) Lactoferrin (↓) Lysozyme (=) **LL-37** (**=**) sIgA (=)	Kunz et al., *Eur. J. Appl. Physiol.* 2015 [70]
20 marathon runners (M, 21 ± 2 y), 20 sedentary controls (M, 20 ± 5 y)	Marathon runners vs. sedentary individuals: **LL-37** (**↓**) **hβD-2** (**↓**)	Usui et al., *J. Sports Med. Phys. Fitness* 2012 [71]

^1^ Concentrations refer to whole saliva; AMP levels were measured using the ELISA method, except for [61], where a combination of ELISA and liquid chromatography–tandem mass spectroscopy (LC-MS/MS) was used. When antimicrobial proteins were measured, their variations were reported. AMPs are highlighted in bold. y—years; M—males; F—females; VO_2max_—maximal oxygen consumption; hNP—human neutrophil peptide; sIgA—secreted immunoglobulin A; hβD—human β-defensin.

**Table 4:** Effects of diet supplementation on salivary AMP levels.

Supplement	Participants and Study Design	Observed Changes in Salivary AMPs Levels ^1^	References
**Vitamin D**	39 athletes (M, 20 ± 2 y) were daily supplemented with vitamin D_3_ (5000 IU, n = 20) or placebo (n = 19) for 14 wk during the winter training period. Saliva sampling: at t_0_, 7, and 14 wk	Supplemented vs. placebo: sIgA (=) lactoferrin (=) lysozyme (=) **LL-37 (=)** SR of sIgA (↑) **LL-37 (↑)**	He et al., *J. Sports Sci.* 2016 [78]
	149 subjects (75 M, 74 F, 19 ± 2 y) completed 12 wk of basic military training with supplementation of vitamin D_3_ (1000 IU) + 2000 mg calcium/d (n = 73) or placebo (n = 76). Saliva sampling: pre-, during (4 and 8 wk), and post-training (12 wk)	Supplemented vs. placebo: SR of sIgA (↑) **LL-37 (↑)** only in M	Scott et al., *Scand. J. Med. Sci. Sports* 2019 [79]
	80 F (20–30 y) divided into two groups: low level (<0.4 IU/mL, n = 40) and high level of serum vitamin D (>1.2 IU/mL, n = 40)	High vs. low vit D levels: **LL-37 (↑)**	Alhelfi et al., *Al-Kindy Col. Med. J.* 2023 [80]
	Two groups of subjects (18–40 y): caries-free (n = 105, 38 M, 67 F) and caries-active (n = 272, 100 M, 172 F)	Caries-free vs. caries-active: vitamin D (↑) **LL-37 (=)**	Nireeksha et al., *BMC Oral Health* 2024 [81]
**Fermented milk and probiotics**	42 M marathonists ingested probiotic *Lactobacillus fermentum* (40 billion CFU/d, n = 20, 40 ± 9 y) or a placebo (n = 22, 40 ± 10 y) for 30 d pre-marathon. Saliva sampling: before and after supplementation, immediately, 72 h, and 14 d post-marathon	Supplemented vs. placebo: sIgA (↑) **hNP 1 (↑)** **LL-37 (=)** lactoferrin (=) lysozyme (=)	Vaisberg et al., *Nutrients* 2019 [82]
	60 children (26 M, 34 F, 13–15 y) were randomly assigned to intervention or control groups. Supplemented for 1 y with *L. paracasei* (6 billion CFU/d) or a placebo for 6 mth Saliva sampling: at baseline and every 3 mth for 1 y	Supplemented vs. placebo: **hNP 1–3 (↑)**	Wattanarat et al., *BMC Oral Health* 2015 [83]
	A cohort of children (1–5 y) without early childhood caries, with early childhood caries, and with severe early childhood caries were randomly assigned to three groups: (i) placebo (n = 86); (ii) daily probiotic (n = 89, 3 × 10^7^ CFU of *L. paracasei*/d), and triweekly probiotic (n = 93, 3 × 10^7^ CFU of *L. paracasei*). Saliva sampling: at baseline, 6 mth, and 12 mth	Daily and weekly vs. placebo: **hNP 1–3** (**↑**) between baseline and month 12 only for children with severe early childhood caries	Wattanarat et al., *Clin. Oral Investig.* 2021 [84]
	42 children were randomly assigned to 2 groups: (i) 11 M and 10 F (2.9 ± 0.3 y) were given a placebo, 11 M and 10 F (3.0 ± 0.3 y) were given *L. rhamnosus* supplemented milk (1.5 billion CFU/d). Saliva sampling: at baseline and end of the study (10 mth)	Supplemented vs. placebo: **hβD-3 (↓)**	Sandoval et al., *Clin. Oral Investig.* 2021 [85]
**Carbohydrates vs. proteins**	Master-aged triathletes (n = 16, 35–60 y) were randomly assigned to ingest, during a 10 wk endurance training, either a hydrolyzed beef protein (n = 8) or a non-protein isoenergetic carbohydrate (n = 8). Saliva sampling: before and after performing an incremental endurance test to exhaustion, pre- and post-intervention	Baselines: **hNP 1–3 (=)** Only for the protein group, post- vs. before exercise: **hNP1–3 (↓)**	Naclerio et al., *J. Am. Coll. Nutr.* 2019 [86]
	27 recreationally physically active subjects (n = 9/treatment) were randomly assigned to 1 of 3 groups: (i) hydrolyzed beef protein (26 ± 5 y), (ii) whey protein (28 ± 5 y), (iii) non-protein isoenergetic carbohydrate (24 ± 7 y). Products were taken once a day for 8 wk during a resistance training program	Baselines: **hNP 1–3 (=)** Only for the beef protein group, post- vs. before exercise: **hNP1–3 (↓)**	Naclerio et al., *Eur. J. Appl. Physiol.* 2017 [87]
**Vitamin A** (retinoic acid)	69 subjects: 34 RA users (16 M, 18 F, 24 ± 4 y) and 35 controls (17 M, 18 F, 25 ± 3 y)	RA vs. controls: **hβD-2 (↓)** **hβD-1 (=)** **hβD-3 (=)**	Atalay et al., *J. Periodontol.* 2023 [88]

^1^ All the studies were performed using unstimulated whole saliva; AMP levels were analyzed using the ELISA method. When antimicrobial proteins were measured, their variations were reported. AMPs are highlighted in bold. y—years; d—day; M—males; F—females; IU—international units; wk—week; SR—secretion rate; sIgA—secreted immunoglobulin A; CFU—colonies forming unit; hNP—human neutrophil peptide; mth—month; RA—retinoic acid; hβD—human β-defensin.

**Table 5:** Influence of tobacco smoking on salivary AMP levels.

Participants and Study Design	Changes in Salivary AMP Levels ^1^	References
40 healthy non-smoker subjects (HNS, 31 M, 9 F, 43 ± 10 y), 40 healthy smokers (HS, 40 M, 0 F, 45 ± 10 y), 40 non-smokers with periodontal disease (PNS, 35 M, 5 F, 45 ± 9 y), 40 smokers with periodontal disease (PS, 40 M, 0 F, 45 ± 9 y)	HS vs. HNS and PS vs. PNS: **LL-37 (↓)**	Kzar et al., *Biomed. Res. Int.* 2023 [40]
69 patients with chronic periodontitis (31 M, 38 F, 43 ± 10 y). Two groups were defined based on the salivary concentration of cotinine (marker of smoking): high (≥8 ng/mL, 14 patients) and low (<8 ng/mL, 55)	High vs. low cotinine: **LL-37 (↓)**	Takeuchi et al., *J. Periodontol.* 2012 [36]
180 children: 90 passive smoking-exposed (PSE, 52 M, 38 F, 9.4 ± 1.6 y) and 90 passive smoking non-exposed (PSU, 47 M, 43 F, 9.0 ± 1.7 y)	PSE vs. PSU: **LL-37 (↓)**	Karsiyaka Hendek et al., *Int. J. Paediatr. Dent.* 2019 [99]
41 individuals: HNS (5 M, 6 F, 53 ± 13 y); PNS (5 M, F 5, 51 ± 18 y); HS (5 M, F 5, 33 ± 11 y); PS (5 M, F 4, 51 ± 15 y)	HNS vs. HS: 63 AMPs and proteins were measured. AMPs: **adrenomedullin (=)**, **dermcidin (=)**, **different hβDs (=)**, **hNP 1 (=)**, **LL-37 precursor (=)**, **neuropeptide Y (=)** PS vs. PNS: **Adrenomedullin (↑)** Eosinophil peroxidase (↑) Three histones (↑) Myeloperoxidase (↑) **hNP 1 (↑)**	Grant et al., *J. Innate Immun.* 2019 [25]

^1^ All the studies were performed using unstimulated whole saliva except for [25] (stimulated whole saliva); AMP levels were analyzed using the ELISA method except for [25] (selected monitoring reaction mass spectroscopy); their variations were reported when antimicrobial proteins were measured. AMPs are highlighted in bold. y—years; M—males; F—females; hNP—human neutrophil peptide; hβD—human β-defensin.

**Table 6:** Influence of psychological stress on salivary AMP levels.

Participants and Study Design	Changes in Salivary AMP Levels ^1^	References
75 army students were classified as not stressed (22 M, 4 F, 24 ± 5 y) or stressed (14 M, 35 F, 22 ± 2 y), according to a stress system inventory	Stressed vs. non-stressed: **hβD-2 (=), hβD-3 (=)**	Forte et al., *J. Oral Pathol. Med.* 2010 [108]
15 adults (60 ± 8 y). Saliva sampling: before and immediately after performing yoga or resting for 90 min	Pre- vs. post-yoga session: **hβD-2 (↑)**	Eda et al., *Eur. J. Appl. Physiol.* 2013 [109]
4 veteran (4 M, 51–53 y) and 4 rookie (3 M, 1 F, 37–45 y) ISS crew members in a 6 mth mission to the ISS, 6 ground-based control subjects (5 M, 1 F, 27–42 y). Saliva sampling: at 180 and 60 d before launch, 10 and 90 d of flight, and 1 d before return	ISS crew vs. controls: sIgA (↑) Lysozyme (↑) **LL-37 (↑)** Lactoferrin (=) **hNP 1–3 (=)**. Rookies vs. veterans: sIgA (↓) Lysozyme (↑) **LL-37 (↑)** Lactoferrin (=) **hNP 1–3 (=)**	Agha et al., *J. Appl. Physiol.* 2020 [110]
4 M and 4 F (23 ± 2 y) completed 2 exercise trials (45 min of running at 75% VO_2max_) after a normal night of sleep (CON) and after a night without sleep (WS). Saliva sampling: before, immediately after, and 1 h after exercise	WS vs. CON: **hNP 1–3 (=)** sIgA (=) Lysozyme (=) Lactoferrin (=) **LL-37 (=)**	Gillum et al., *J. Strength Cond. Res.* 2015 [69]
44 students (18–25 y). Saliva sampling: before and immediately after the examination	Post- vs. pre-examination **:** **Neuropeptide Y** **(↑** **)**	Semsi et al., *Mol. Aspects Med.* 2023 [111]

^1^ All the studies were performed using unstimulated whole saliva except for [109] (stimulated whole saliva); AMP levels were analyzed using the ELISA method; their variations were reported when antimicrobial proteins were measured. AMPs are highlighted in bold. y—years; M—males; F—females; hβD—human β-defensin; ISS—International Space Station; sIgA—secreted immunoglobulin A; mth—month; VO_2max_—maximal oxygen consumption.
